# Treatment of duodenal lipoma with robotic-assisted transverse duodenotomy: A case report of novel approach

**DOI:** 10.1016/j.ijscr.2021.106366

**Published:** 2021-09-06

**Authors:** Logan D. Glosser, Conner V. Lombardi, Hanna M. Knauss, Wade Hopper, Abdullah Alalwan, Stephen Stanek

**Affiliations:** a University of Toledo College of Medicine and Life Sciences, 3000 Arlington Ave, Toledo, OH 43614, USA; b University of Toledo College of Medicine and Life Sciences, Department of General Surgery, 3000 Arlington Ave, Toledo, OH 43614, USA; c Edward via College of Osteopathic Medicine, 350 Howard St, Spartanburg, SC 29307, USA; d Toledo Promedica, Dept. of General Surgery, 2142 N Cove Blvd, Toledo, OH 43606, USA

**Keywords:** PCP, Primary Care Physician, EGD, Esophagogastroduodenoscopy, ICG, Indocyanine Green, GI, gastrointestinal, IBS, irritable bowel syndrome, Duodenal lipoma, Transverse duodenotomy, Robotic surgery, Gastrointestinal tumors, Constipation, Case report

## Abstract

**Introduction and importance:**

Lipomas are the third most common benign tumor of the gastrointestinal (GI) tract, typically occurring in the colon or small intestine. Less than 100 cases of symptomatic duodenal lipomas have been reported. Symptoms include non-specific upper GI complaints of heartburn, fullness, or abdominal pain. This report highlights the rarity of symptomatic duodenal lipomas, lack of specific treatment guidelines, and adds to surgical literature a new treatment approach.

**Case presentation:**

A 53-year-old Caucasian woman presented with 2-year history with main concerns for early satiety and constipation. CT scan with contrast of the abdomen and pelvis demonstrated a duodenal mass. Differential diagnosis included duodenal lipoma versus stricture, and IBS. Subsequent EGD revealed a 4 cm transverse duodenal submucosal mass. Endoscopic removal was deemed too great a risk of bleeding. Pre-operatively, the patient expressed frustration as the patient was tolerating only a liquid diet with one bowel movement weekly. Treatment with robotic assisted transverse duodenotomy was performed, with final pathology of benign lipomatous tissue. Post-operatively the patient had immediate relief of symptoms which persisted at 2-week and 4-month follow-ups.

**Clinical discussion:**

This case demonstrates 3 primary learning points. First, duodenal lipomas should be included in the differential of vague upper GI symptoms. Second, we propose that surgeons consider treatment of duodenal lipomas utilizing robotic assisted approach. Third, we document the first robotic-assisted transverse duodenotomy for duodenal lipomas.

**Conclusion:**

Clinicians should consider duodenal lipoma for patients with vague abdominal symptoms. We present a case of successful treatment with robotic-assisted transverse duodenotomy.

## Highlights

• Only a few cases of symptomatic duodenal lipoma have been reported.
• No specific treatment guidelines exist for duodenal lipomas.
• First-reported treatment of duodenal lipoma via robotic-assisted transverse duodenotomy

## 1 Introduction

Duodenal lipomas are a rare benign tumor accounting for 4% of gastrointestinal lipomas. Duodenal lipomas are typically asymptomatic and often found incidentally [1]. Lipomas can cause obstructive symptoms if they increase in size, particularly over 2 cm diameter, presenting with pain, early satiety, and fullness [2], [3]. With less than 100 cases of symptomatic duodenal lipomas reported, there is no standardized treatment [2]. Limited management approaches are documented in the literature, including endoscopic, laparoscopic, and open approaches to resection with duodenectomy or, more rarely, duodenotomy [2]. As with many fields of medicine, robotic procedures have become increasingly popular due to fewer surgical complications and accelerated recovery times. We present a case from an academic hospital in which a patient with symptomatic duodenal lipoma was successfully treated with bowel-sparing robotic assisted laparoscopic transverse duodenotomy. This case report has been reported in line with the SCARE Criteria [4].

## 2 Presentation of case

**Fig. 1:**
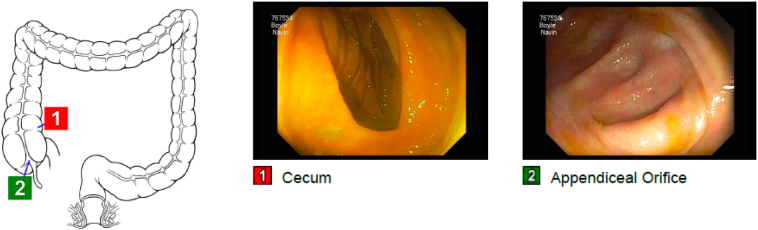
Colonoscopy with removal of 3 sessile polyps 5–6 mm in size located in the rectum.

**Fig. 2:**
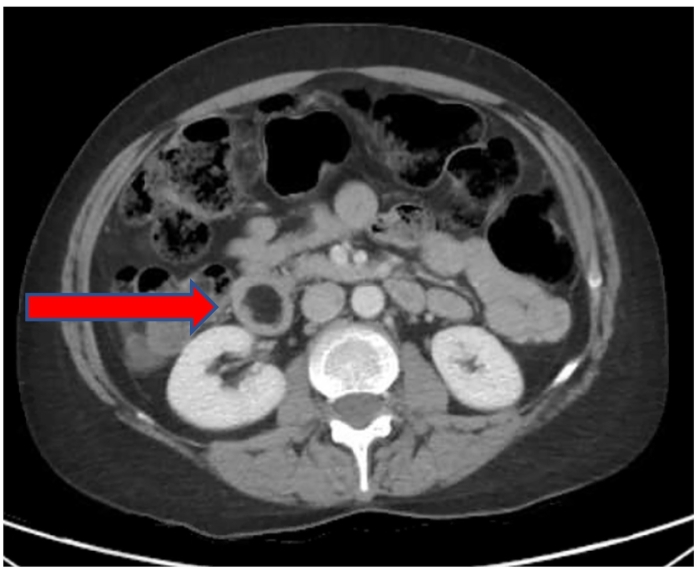
CT scan revealing duodenal mass anterior to right kidney.

**Fig. 3:**
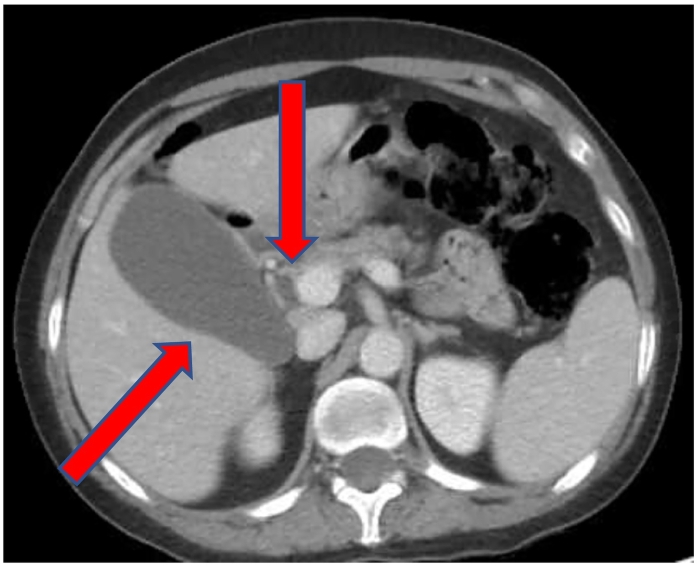
CT scan: Enlarged gallbladder overlying duodenal lipoma.

**Fig. 4:**
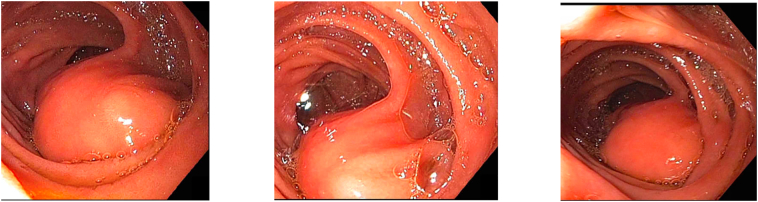
EGD: Large 4 cm submucosal mass in the third segment of the duodenum, D3.

**Fig. 5:**
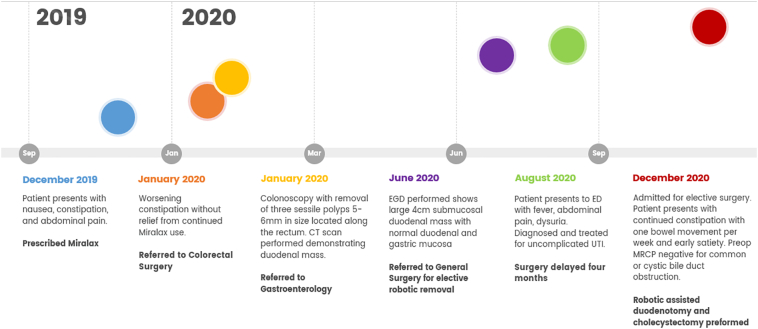
Patient timeline of events from initial encounter to final procedure.

A 53-year-old Caucasian non-obese female self-presented to the primary care provider (PCP) with a 1-year history of worsening constipation and inability to tolerate food. Past medical history was significant for anxiety, depression, attention deficit hyperactivity disorder, fibromyalgia, and hiatal hernia. Past surgical history significant for umbilical hernia repair 5 years prior with multiple prior lipoma resections. Her drug history and psychosocial history were unremarkable. Her family history did not include any relevant genetic information. The patient reported being single not-married, employment with child-care services, and living independently. The patient had a prior 35 pack year smoker (quit 1 year prior to surgery), did not use alcohol, and no known drug allergies. Physical exam showed abdominal tenderness with mild epigastric discomfort and tenderness to palpation. The PCP prescribed oral polyethylene glycol, 17-gram packet once daily, which provided no relief. Oral lubiprostone 24 mcg capsule was added once daily, but at 3 months later the patient reported spaghetti-string stools and no relief of symptoms, thus was referred to colorectal surgery. A colonoscopy with polyp removal (Fig. 1) was performed without relief of symptoms. The patient reported no relief with poly-ethylene glycol and lubiprostone combination, thus she was given a trial of plecanatide 3 mg tablet oral three times Daily. A subsequent CT scan of the abdomen and Pelvis was ordered and demonstrated a mass obstructing approximately 75% of the duodenal lumen (Fig. 2) laying abut to an enlarged gallbladder (Fig. 3). The differential diagnosis included duodenal lipoma, duodenal stricture, and irritable bowel syndrome. Gastroenterology was consulted, and 6 months later performed an Esophagogastroduodenoscopy (EGD) confirming an intraluminal duodenal mass (Fig. 4). The case was discussed for possible endoscopic submucosal resection; however, the risk of bleeding by this approach was too great. General surgery was brought on board and scheduled a robotic assisted laparoscopic mass excision, as displayed in the timeline (Fig. 5).

Unfortunately, the patient contracted a urinary tract infection and the surgery was delayed another 4 months. By the time of presentation for surgery, the patient reported only being able to have pureed foods and soups for over 1 year with less than 1 bowel movement per week. The patient reported frustration with her lack of improvement over the past year and delay from diagnosis to treatment. The patient had poor compliance with laxative therapies with polyethylene glycol and plecanatide as she was embarrassed by constant uncontrollable flatus. Preadmission labs with CBC, BMP, Urine Analysis, PT, and PTT were all within normal limits.

On admission, a preoperative magnetic resonance cholangiopancreatography (MRCP) was negative for common or cystic bile duct obstruction. Antibiotic prophylaxis with IV cefazolin 2000 mg/50 mL in sodium chloride 0.9% was initiated. The patient was brought to the operating room for the procedure. The Operator was a junior faculty with 5 years of post-residency Da Vinci robotics experience at a university-affiliated hospital. An endoscope was advanced into the stomach and further into the duodenum to visualize the mucosa. After confirming the location of the tumor, the Da Vinci robotic device was attached with the patient supine in the reverse Trendelenburg position and slightly turned to the left.

A chronically inflamed gallbladder was visible along with multiple adhesions to the hepatic flexure. The adhesions were taken down with blunt and sharp dissection. The stomach and transverse colon were identified, and access to the lesser sac was obtained. The gastrocolic ligament was taken down with blunt and sharp dissection. Dissection was carried to the hepatic flexure and the right colon was retracted inferiorly. The stomach was retracted medially and tacked to the falciform ligament using 3-0 V-Loc. Kocherization of the duodenum was achieved.

The duodenal mass was identified and injected with 2 mL of Indocyanine Green (ICG) for visualization using Firefly Fluorescence imaging with the Da Vinci Robot. The whole duodenum was enhanced with ICG; therefore, in addition to the planned procedure, an intraoperative ultrasound was used to confirm the location of the mass. At this point a transverse duodenotomy was begun. After surgical dissection through the seromuscular layer overlying the mass, the mass was detached from the mucosal layer and dissected out from the duodenal wall. The transverse duodenotomy was closed with mucosal and full thickness closures with 3-0 V-loc sutures. A second layer of lembert suture was placed to secure the repair using 3-0 V-loc. Omental flaps were tacked on top of the repair using 3-0 V-loc. Prior to closure a robotic cholecystectomy was also performed. Pathology of the excised mass confirmed benign lipomatous soft tissue, procuring a good prognosis. Totally operative time was 4 h 18 m, 3800 mL crystalloid was administered with estimated blood loss of 30 mL and 785 mL urine output. This is the “first in-human” robotic assisted transverse duodenotomy for duodenal lipoma resection.

DVT/VTE prophylaxis was initiated post-operatively with enoxaparin 40 mg injections daily. Pain was controlled with acetaminophen 1000 mg IV q8 and morphine 4 mg IV q6 PRN. Oral pain medications were not administered to protect bowel function. The patient did not have any postoperative complications or adverse events. The patient obtained the expected clinical outcome with complete relief of obstructive symptoms. The patient was kept NPO for 2 days post-operatively to allow healing of the duodenal resection site. On post-op day 3 the patient tolerated regular adult diet for breakfast and lunch with relief of the pre-operative early satiety with meals. The patient was subsequently discharged home within the expected time frame of 2–3 days post-operatively. The patient was discharged with scheduled polyethylene glycol 17 g packet once daily in the morning for 2 weeks, after which it would be PRN. At the 2-week appointment the patient stated she did not require use. The patient confirmed relief of symptoms at 2-week and 4-month in person postoperative follow-up appointments, without residual constipation or early satiety. The patient self-reported adherence to 2-week avoidance of heavy lifting >20 pounds. The patient self-reported tolerating a normal adult diet without restriction and regular bowel movements every 2–3 days, without the need for additional medications or laxatives. At this point the patient did not require any future surveillance for the lipoma. No post-discharge labs were indicated nor performed.

## 3 Discussion

Lipomas are the third most common benign tumor affecting the Gastrointestinal (GI) tract [5]. They occur most commonly in the colon, followed by the small intestine and the duodenum [5]. Duodenal lipomas are extremely rare, accounting for only 4% of all GI tumors [1], [5]. In a systematic review with search of ‘duodenal lipoma’ on PubMed from 1948 to 2016, only 59 cases of duodenal lipoma were reported [2]. Duodenal lipomas typically present as a single round mass in the submucosa of the second portion of the duodenum. Only 16% of duodenal lipomas present in the third part of the duodenum, making our case even more rare [2], although literature on duodenal lipomas is scarce. The average size recorded is 4.1 cm, with 80% of symptomatic duodenal lipomas having a diameter greater than 2 cm [2]. Duodenal lipomas are classified as either submucosal, as in our case, or subserosal. They are also classified by their gross appearance as sessile or pedunculated [5].

Although rare, most duodenal lipomas are asymptomatic with diagnoses made incidentally via routine endoscopy or GI surgery [3]. Due to the low chance of malignancy, asymptomatic duodenal lipomas do not require treatment [3]. Symptomatic duodenal lipomas initially present with nonspecific upper GI complaints including abdominal discomfort, pain, and fullness [3]. More severe cases may present with GI bleeding, ulceration, gastric obstruction, and rarely, intussusception [6]. Many of these symptoms can be attributed to the obstructive nature of the mass itself. Our patient presented with a 1-year history of increasing constipation with bouts of nausea and abdominal pain.

Due to duodenal lipomas' nonspecific symptomatic presentation, further clinical investigation is necessary for differential diagnosis. CT, MRI, and ultrasound are used to identify duodenal lipomas; however, imaging modalities are mostly used in conjunction with endoscopy to further delineate origin and pathology [7], [8]. Imaging for duodenal lipomas includes CT showing a characteristic low density, MRI with signals on T1 and T2 weighted images, and esophageal ultrasound demonstrating a homogenous hyperechoic appearance [7], [8]. Biopsy of the mass is typically used to confirm the composition of the tumor [2]. In our case, the large, obstructive nature of the duodenal mass led to GI symptoms warranting an abdominal CT, which revealed a suspected duodenal lipoma. The diagnosis of duodenal lipoma was confirmed on pathology of the excised mass.

There is no standard of care to treat symptomatic duodenal lipomas. Treatment with endoscopic excision is limited to small-pedunculated tumors, whereas larger or more complex tumors carry a higher risk of perforation and bleeding for which laparoscopic or open treatment is indicated [9]. Endoscopic excision was considered but this option was not pursued due to the risk of bleeding.

The most common documented surgical intervention for duodenal lipomas is laparoscopic trans-duodenal resection, otherwise known as duodenectomy [10]. Less common treatment options include partial duodenectomy with anastomoses, duodenotomy, and rarely gastric bypass surgery. There are currently no randomized studies that definitively compare duodenotomy and duodenectomy. Duodenotomy may be chosen over duodenectomy in cases that are nontraumatic or involve solitary masses [11]. In duodenotomy, transverse closure of the duodenum is often preferred over longitudinal suturing in order to preserve luminal diameter, avoid wound leakage, and discourage strictures from forming [12]. Complications of transverse duodenectomy include suture dehiscence, ileus, fistulation, and higher neoplastic recurrence compared to duodenectomy [11].

Robotic and laparoscopic approaches are increasingly popular due to their minimally invasive nature, low complication rate, and accelerated recovery times. Our case demonstrates a successful minimally invasive approach, with no post-surgical complications or adverse events. The patient immediately felt relief with her first meal and reported continued relief of obstructive symptoms 4 months post-operation and expressed satisfaction with her care. These study findings are limited as we report a single patient; however, it is significant due to the lack of symptomatic duodenal lipomas reported in the literature. Transverse duodenotomy requires high precision to ensure the correct plane, thus, to be done with a robotic approach the operator must be experienced. Alternatives to this approach include an open surgical approach, endoscopic approach, or duodenectomy.

This case demonstrates 3 primary learning points. First, duodenal lipomas should be included in the differential diagnosis of vague upper GI symptoms. Second, we propose that surgeons consider treatment of duodenal lipomas utilizing robotic assisted approach. Third, our experience offers evidence of using less invasive transverse duodenotomy for treatment, rather than duodenectomy.

## 4 Conclusion

Duodenal lipomas are rare tumors that are typically benign and asymptomatic but can cause significant morbidity. Symptomatic duodenal lipomas that present with signs of obstruction or bleeding require procedural intervention. There is currently no standard of care for symptomatic duodenal lipomas necessitating surgical removal. Our case adds a new perspective to the literature suggesting that further consideration should be given to the robotic-assisted bowel-sparing laparoscopic transverse duodenotomy.

## Ethical approval

N/a

## Sources of funding

This research did not receive any specific grant from funding agencies in the public, commercial, or not-for-profit sectors.

## CRediT authorship contribution statement

Logan D. Glosser: Investigation, Writing – Original Draft, Writing – Review & Editing, Project administration. Conner Lombardi: Investigation, Writing – Original Draft, Visualization. Hanna Knauss: Writing – Review & editing. Abdullah Alalwan: Conceptualization. Wade Hopper: Validation, Writing Review & Editing. Stephen Stanek: Supervision, Writing – Review & editing.

All authors read and approved the final version to be published.

## Guarantor

Logan D. Glosser. University of Toledo College of Medicine and Life Science, Toledo, OH, USA. Phone:419-705-6757

## Research registration Unique Identifying Number

researchregistry7090

https://www.researchregistry.com/register-now#home/registrationdetails/6123e6f247c3c9001e7dc5ce/

## Consent

Written informed consent was obtained from the patient for publication of this case report and accompanying images. A copy of the written consent is available for review by the Editor-in-Chief of this journal on request.

## Provenance and peer review

Not commissioned, externally peer-reviewed.

## Declaration of competing interest

None declared.
